# Factors associated with the surgical outcomes of Baerveldt glaucoma implant for open-angle glaucoma, an age-related eye disease

**DOI:** 10.1038/s41598-021-04570-4

**Published:** 2022-01-25

**Authors:** Satoshi Iraha, Yuji Takihara, Yui Urahashi, Takahiro Watanabe, Kenichi Nakamura, Mai Urahashi, Fumika Watanabe-Kitamura, Kei-Ichi Nakashima, Eri Takahashi, Sachi Kojima, Hidenobu Tanihara, Toshihiro Inoue

**Affiliations:** 1 Department of Ophthalmology, Faculty of Life Sciences, Kumamoto University, 1-1-1 Honjo, Chuo-ku, Kumamoto, 860-8556 Japan; 2 Department of Ophthalmology, National Sanatorium Kikuchi Keifuen, Kumamoto, Japan

**Keywords:** Glaucoma, Optic nerve diseases

## Abstract

To identify the factors associated with the surgical outcomes of Baerveldt glaucoma implant (BGI) for open-angle glaucoma (OAG), the medical records of 51 consecutive OAG patients (age, 43–91 years) who underwent BGI were retrospectively reviewed (median follow-up, 21.7 months). Surgical success was defined as the following postoperative intraocular pressures (IOPs, mmHg): (A) 6 ≤ IOP ≤ 21; (B) 6 ≤ IOP ≤ 18; and (C) 6 ≤ IOP ≤ 15 without loss of light perception or additional glaucoma surgery. Univariate analysis showed that age (all criteria), glaucoma type (criterion C), and preoperative IOP (criteria A and B) were the candidate factors (*P* < 0.20). When the patients were divided into two groups according to median age (72 years), the success probability was higher in the older group for criteria B (*P* = 0.047) and C (*P* = 0.02), and the postoperative IOP was lower in the older group 1-year post-surgery (*P* = 0.002). Furthermore, the multivariate Cox proportional hazards model revealed that older age was independently associated with surgical success for criteria B (relative risk [RR], 0.94; *P* = 0.02) and C (RR, 0.94; *P* = 0.01). In conclusion, older age is a factor associated with the surgical success of BGI for OAG.

## Introduction

Open-angle glaucoma (OAG) is an age-related eye disease whose incidence increases with aging^1–3^. The number of patients with glaucoma is rapidly rising and estimated to reach 111.8 million by 2040^4^. OAG significantly contributes to this rapid rise because life expectancy is globally increasing which is brought about by medical advances^5^. In this situation, it is necessary to improve the surgical strategies for OAG by identifying the factors associated with the surgical outcomes.

Although trabeculectomy with mitomycin C (MMC) is a popular glaucoma surgery, implant surgeries using a glaucoma drainage device (GDD), such as Baerveldt glaucoma implant (BGI), Ahmed glaucoma valve (AGV), and Molteno implant, have been widely performed in recent years. GDD was initially used for refractory glaucoma such as neovascular glaucoma. However, recent prospective multicenter trials have suggested the efficacy and safety of GDD for many glaucoma types, including OAG^6–10^. After Molteno introduced GDD in 1969, many studies have shown the treatment outcomes and factors associated with the surgical outcomes of GDD^11–18^. However, most of the previous studies included refractory glaucoma, whose surgical outcomes of GDD are different from those for OAG. Importantly, the factors associated with the surgical outcomes of BGI for OAG remain unclear.

This study aims to investigate the factors associated with the surgical outcomes of BGI for OAG using the Kaplan–Meier survival curve and Cox proportional hazards model analysis. The results in this study would facilitate the improvement of the surgical strategies for OAG in an aging society.

## Results

### Baseline characteristics and probability of success of all eyes

In the present study, 51 eyes of 51 patients with OAG were examined with a median follow-up of 21.7 months. The preoperative characteristics of all eyes are described in Table 1. The mean (standard deviation [SD]) age at BGI surgeries was 71.6 (± 11.0) years old. The mean (SD) preoperative intraocular pressure (IOP) was 29.4 (± 7.1) mmHg. The mean (SD) preoperative number of glaucoma medications was 4.2 (± 0.7).

**Table 1:** Preoperative characteristics of all 51 patients with OAG.

Characteristic	Value
	(n = 51)
**Age, years**	
Mean (SD)	71.6 (11.0)
Range	43–91
**Gender, No. (%)**	
Male	37 (73%)
Female	14 (27%)
**Eye laterality, No. (%)**	
Right	20 (39%)
Left	31 (61%)
**Glaucoma type, No. (%)**	
POAG	19 (37%)
EXG	32 (63%)
**Hypertension, No. (%)**	21 (41%)
**Diabetes mellitus, No. (%)**	9 (18%)
**Number of previous glaucoma filtration surgeries, No. (%)**	
0	2 (4%)
1	28 (55%)
2	19 (37%)
3	2 (4%)
**Previous cataract surgery, No. (%)**	38 (75%)
**Preoperative IOP, mmHg**	
Mean (SD)	29.4 (7.1)
**Preoperative glaucoma medications, No**	
Mean (SD)	4.2 (0.7)
**BCVA, logMAR**	
Mean (SD)	0.55 (0.73)
**Automated perimetry program 24-2**	
Eyes, No	37
MD, mean (SD), dB	− 20.7 (7.5)
**Automated perimetry program 10-2**	
Eyes, No	9
MD, mean (SD), dB	− 27.7 (6.1)
**Location of plate implantation, No. (%)**	
Superotemporal	33 (65%)
Inferotemporal	18 (35%)
**Location of tube implantation, No. (%)**	
Anterior chamber	48 (94%)
Vitreous	3 (6%)

*OAG* open-angle glaucoma, *SD* standard deviation, *POAG* primary open-angle glaucoma, *EXG* exfoliation glaucoma, *IOP* intraocular pressure, *BCVA* best-corrected visual acuity, *logMAR* logarithm of the minimum angle of resolution, *MD* mean deviation.

**Figure 1:**
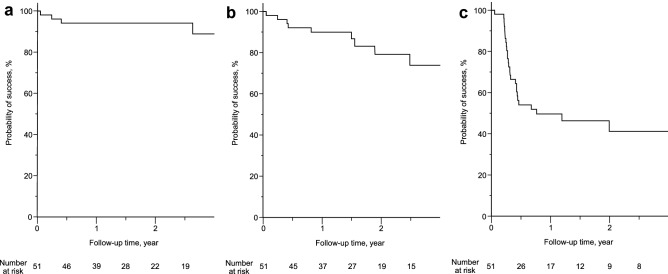
The Kaplan–Meier survival curves for the success probability of BGI of all 51 OAG eyes. (**a**) Criterion A (6 ≤ IOP ≤ 21 mmHg). (**b**) Criterion B (6 ≤ IOP ≤ 18 mmHg). (**c**) Criterion C (6 ≤ IOP ≤ 15 mmHg). The number of eyes at risk is indicated at the bottom. BGI, Baerveldt glaucoma implant; OAG, open-angle glaucoma; IOP, intraocular pressure.

As described in the Methods section, surgical success was defined as the following IOPs: 6–21 mmHg (criterion A), 6–18 mmHg (criterion B), and 6–15 mmHg (criterion C). The Kaplan–Meier survival curve of the surgical outcomes of all 51 eyes for the three criteria is shown in Fig. 1. The success probability 1 and 2 years after BGI was 94.1% for criterion A. The success probabilities 1 and 2 years after BGI were 89.9% and 79.2% for criterion B, respectively. The success probabilities 1 and 2 years after BGI were 49.6% and 41.2% for criterion C, respectively. The reasons for surgical failure were hypotony in one eye, BGI removal in one eye, and elevated IOP in 2, 7, and 26 eyes for criteria A, B, and C, respectively. Elevated IOP of 1, 6, and 19 eyes for criteria A, B, and C, respectively, were managed by glaucoma medications. One eye needed bleb revision, micropulse transscleral cyclophotocoagulation, and additional BGI surgery after surgical failure for criteria A, B, and C due to elevated IOP. No surgical failure due to loss of light perception was observed.

### Univariate analysis for candidate factors associated with the surgical outcomes

To find the candidate factors associated with the surgical outcomes of BGI for OAG, we analyzed the association between the surgical outcomes of BGI and the following factors shown in Table 2 using the univariate Cox proportional hazards model: age, gender, glaucoma type, preoperative IOP, previous cataract surgery, the number of previous glaucoma filtration surgeries, eye laterality, hypertension, diabetes mellitus, and location of plate and tube implantation. When the factors that had a *P* value < 0.20 were regarded as the candidate factors, the analysis showed that age (all criteria), glaucoma type (criterion C), and preoperative IOP (criteria A and B) were the candidate factors associated with the surgical outcomes of BGI.

**Table 2:** Univariate analysis of candidate factors associated with the surgical outcomes of BGI for OAG by the Cox proportional hazards model.

Variable	*P* value		
	Criterion A	Criterion B	Criterion C
	6 ≤ IOP ≤ 21 mmHg	6 ≤ IOP ≤ 18 mmHg	6 ≤ IOP ≤ 15 mmHg
Age	**0.10**	**0.007**	**0.002**
Gender	0.84	0.70	0.53
Glaucoma type (POAG/EXG)	0.59	0.28	**0.04**
Preoperative IOP	**0.08**	**0.08**	0.48
Previous cataract surgery	0.89	0.59	0.97
Number of previous glaucoma filtration surgeries	0.30	0.30	0.32
Eye laterality	0.59	0.77	0.82
Hypertension	0.66	0.27	0.52
Diabetes mellitus	0.66	0.68	0.72
Location of plate implantation (superotemporal/inferotempral)	0.43	0.50	0.58
Location of tube implantation (anterior chamber/vitreous)	0.48	0.53	0.38

*BGI* Baerveldt glaucoma implant, *OAG* open-angle glaucoma, *IOP* intraocular pressure, *POAG* primary open-angle glaucoma, *EXG* exfoliation glaucoma.

*P* values < 0.20 are shown in boldface.

### Comparison of cumulative probability of success

**Figure 2:**
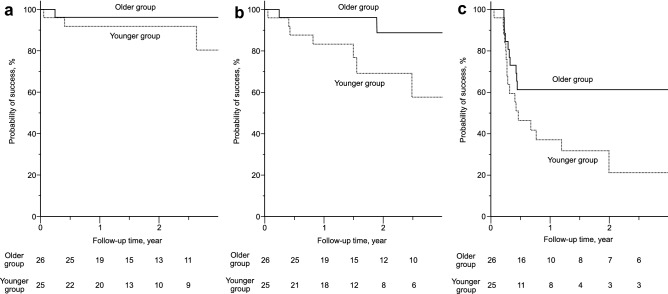
Comparison of the Kaplan–Meier survival curves for the success probability of BGI between patients with OAG aged < 72 years (younger group, dotted line) and those aged ≥ 72 years (older group, solid line). (**a**) Criterion A (6 ≤ IOP ≤ 21 mmHg), *P* = 0.27 by the log-rank test. (**b**) Criterion B (6 ≤ IOP ≤ 18 mmHg), *P* = 0.047 by the log-rank test. (**c**) Criterion C (6 ≤ IOP ≤ 15 mmHg), *P* = 0.02 by the log-rank test. The number of eyes at risk in each group is indicated at the bottom. BGI, Baerveldt glaucoma implant; OAG, open-angle glaucoma; IOP, intraocular pressure.

The results in Table 2 suggest that age has the highest ability to predict the surgical outcomes of BGI for OAG. Thus, the patients were divided into two groups according to the median age of the patients in this study (72 years) to investigate in detail the predictive ability of age. Table 3 shows the comparison of the baseline characteristics of the patients between those aged < 72 years (younger group) and those aged ≥ 72 years (older group). In addition to an age difference, the ratio of patients diagnosed as having exfoliation glaucoma (EXG) in the older group was significantly higher than that in the younger group (*P* = 0.03). There were no other statistically significant differences between the younger and older groups in terms of preoperative characteristics. The results of Kaplan–Meier survival curve analysis to compare the younger and older groups for criteria A, B, and C are described in Fig. 2. For criterion A, the success probabilities both 1 and 2 years after BGI were 96.2% vs 91.8% in the older vs younger groups, respectively. For criterion B, the success probabilities 1 and 2 years after BGI were 96.2% vs 83.3% and 88.8% vs 69.2% in the older vs younger groups, respectively. For criterion C, the success probabilities 1 and 2 years after BGI were 61.3% vs 37.1% and 61.3% vs 21.2% in the older vs younger groups, respectively. A significantly higher probability of success for criteria B (*P* = 0.047) and C (*P* = 0.02) was observed in the older group by the log-rank test. There was a similar tendency for a higher probability of success in the older group than in the younger group for criterion A (*P* = 0.27).

**Table 3:** Comparison of the baseline characteristics between patients with OAG in the younger and older groups.

Characteristic	Younger group	Older group	*P* value
	(n = 25)	(n = 26)	
**Age, years**			** < 0.0001**^**a**^
Mean (SD)	63.3 (9.1)	79.7 (4.9)	
Range	43–71	72–91	
**Gender, No. (%)**			0.93^b^
Male	18 (72%)	19 (73%)	
Female	7 (28%)	7 (27%)	
**Eye laterality, No. (%)**			0.64^b^
Right	9 (36%)	11 (42%)	
Left	16 (64%)	15 (58%)	
**Glaucoma type, No. (%)**			**0.03**^**b**^
POAG	13 (52%)	6 (23%)	
EXG	12 (48%)	20 (77%)	
**Hypertension, No. (%)**	8 (32%)	13 (50%)	0.19^b^
**Diabetes mellitus, No. (%)**	3 (12%)	6 (23%)	0.47^c^
**Number of previous glaucoma filtration surgeries, No. (%)**			0.24^a^
0	0 (0%)	2 (6%)	
1	13 (52%)	15 (58%)	
2	11 (44%)	8 (31%)	
3	1 (4%)	1 (4%)	
**Previous cataract surgery, No. (%)**	16 (64%)	22 (85%)	0.12^c^
**Preoperative IOP, mmHg**			0.30^a^
Mean (SD)	30.3 (7.1)	28.6 (7.2)	
**Preoperative glaucoma medications, No**			0.15^a^
Mean (SD)	4.3 (0.6)	4.1 (0.7)	
**BCVA, logMAR**			0.85^a^
Mean (SD)	0.66 (0.89)	0.45 (0.55)	
**Automated perimetry program 24–2**			0.53^a^
Eyes, No	19	18	
MD, mean (SD), dB	− 19.9 (7.5)	-21.4 (7.5)	
**Automated perimetry program 10–2**			1.00^a^
Eyes, No	5	4	
MD, mean (SD), dB	− 27.5 (7.0)	-28.0 (5.8)	
**Location of plate implantation, No. (%)**			0.91^b^
Superotemporal	16 (64%)	17 (65%)	
Inferotemporal	9 (36%)	9 (35%)	
**Location of tube implantation, No. (%)**			0.61^c^
Anterior chamber	23 (92%)	25 (96%)	
Vitreous	2 (8%)	1 (4%)	

*OAG* open-angle glaucoma, *SD* standard deviation, *POAG* primary open-angle glaucoma, *EXG* exfoliation glaucoma, *IOP* intraocular pressure, *BCVA* best-corrected visual acuity, *logMAR* logarithm of the minimum angle of resolution, *MD* mean deviation.

^a^Mann–Whitney U test. ^b^Chi-square test. ^c^Fisher's exact test.

*P* values < 0.05 are shown in boldface.

Univariate analysis with the Cox proportional hazards model showed that glaucoma type was also a candidate factor associated with the surgical outcomes of BGI for OAG (Table 2). The Kaplan–Meier survival curves of EXG and primary OAG (POAG) groups were shown in Supplementary Fig. S1. The success probability of BGI was significantly higher in the EXG group than in the POAG group for criterion C by the log-rank test (*P* = 0.03).

### IOP and number of glaucoma medications in the younger and older groups

The IOP levels 6 months, 1 year, and 2 years after BGI between the younger and older groups were compared (Table 4). In agreement with the results in Fig. 2, the postoperative IOP level in the older group tended to be consistently lower than that in the younger group during the follow-up. Furthermore, the IOP levels were significantly lower in the older group than those in the younger group 1 year after BGI (Table 4, *P* = 0.002, Mann–Whitney U test). Compared with the respective baseline levels of each group, both groups showed significantly lower IOP levels 6 months, 1 year, and 2 years after BGI (Table 4, all *P* < 0.01, Wilcoxon signed-rank test with Bonferroni correction) and at the final follow-up (Supplementary Fig. S2, *P* < 0.0001, Wilcoxon signed-rank test).

**Table 4:** IOP during follow-ups after BGI.

IOP, mmHg					
Time	Younger group		Older group		*P* value^a^
	Mean ± SD	Number	Mean ± SD	Number	
Baseline	30.3 ± 7.1	25	28.6 ± 7.2	26	0.30
6 months	15.7 ± 3.7	23	14.2 ± 4.0	26	0.17
1 year	15.8 ± 2.4	22	13.4 ± 3.9	25	0.002
2 years	15.0 ± 3.5	11	14.3 ± 3.8	15	0.24

*IOP* intraocular pressure, *BGI* Baerveldt glaucoma implant, *SD* standard deviation. ^a^Mann–Whitney U test.

The number of glaucoma medications was also compared between the younger and older groups 6 months, 1 year, and 2 years after BGI (Table 5). The number of glaucoma medications in the older group tended to be consistently lower than that in the younger group, but the difference did not reach a statistical significance at all the follow-up time points. Compared with the respective baselines of each group, both groups showed significantly fewer medications 6 months, 1 year, and 2 years after BGI (Table 5, all *P* < 0.01, Wilcoxon signed-rank test with Bonferroni correction).

**Table 5:** The number of glaucoma medications during follow-ups after BGI.

Glaucoma medications, No					
Time	Younger group		Older group		*P* value^a^
	Mean ± SD	Number	Mean ± SD	Number	
Baseline	4.3 ± 0.6	25	4.1 ± 0.7	26	0.15
6 months	1.7 ± 1.5	23	1.2 ± 1.5	26	0.22
1 year	1.7 ± 1.5	22	1.3 ± 1.5	25	0.35
2 years	1.7 ± 1.6	11	1.5 ± 1.8	15	0.74

*BGI* Baerveldt glaucoma implant, *SD* standard deviation.

^a^Mann–Whitney U test.

### Multivariate analysis for factors associated with the surgical outcomes

As shown in Table 2, the candidate factors identified by univariate analysis were age (for criteria A, B, and C), preoperative IOP (for criteria A and B), and glaucoma type (for criteria C). To reveal the independent factors associated with the surgical outcomes of BGI for OAG, a multivariate analysis was performed by the Cox proportional hazards model analysis. The analysis indicated that only age was an independent factor associated with the surgical outcomes in criterion B (relative risk [RR], 0.94; 95% confidence interval [CI], 0.89–0.99; *P* = 0.02) and criterion C (RR, 0.94; 95% CI 0.90–0.99; *P* = 0.01), indicating that older age was associated with a higher probability of success of BGI for OAG (Table 6).

**Table 6:** Multivariate analysis to reveal the factors associated with the surgical outcomes of BGI for OAG using the Cox proportional hazards model.

Variable	Criterion A		Criterion B		Criterion C	
	(6 ≤ IOP ≤ 21 mmHg)		(6 ≤ IOP ≤ 18 mmHg)		(6 ≤ IOP ≤ 15 mmHg)	
	RR (95% CI)	*P* value	RR (95% CI)	*P* value	RR (95% CI)	*P* value
Age, per 1-year increase	0.96 (0.88–1.04)	0.31	0.94 (0.89–0.99)	0.02	0.94 (0.90–0.99)	0.01
Glaucoma type	–	–	–	–	0.66 (0.29–1.55)	0.33
Preoperative IOP, per 1-mmHg increase	1.08 (0.95–1.24)	0.22	1.04 (0.96–1.13)	0.34	–	–

*BGI* Baerveldt glaucoma implant, *OAG* open-angle glaucoma, *IOP* intraocular pressure, *RR* relative risk, *CI* confidence interval.

### Complications

The surgical complications that occurred within the first postoperative month were defined as early postoperative complications, and those that developed 1 month after surgery were defined as late postoperative complications. No significant difference between the groups was observed for all the postoperative complications during each early and late phase (Supplementary Table S1). In eyes with tube cornea touch, one eye needed reformation of the anterior chamber, and one eye needed reformation of the anterior chamber and choroidal effusion drainage. One eye with tube occlusion needed release of tube occlusion. In this eye, scleral patch exposure occurred, and BGI was removed. In another eye, blebitis was suspected, which was resolved by additional antibiotic eye drops. Although Shin et al.^19^ have reported that older age is one factor for choroidal detachment after GDD surgery, no significant difference was found between the younger and older groups in this study.

## Discussion

This study aims to reveal the factors associated with the surgical outcomes of BGI for OAG. The univariate Cox proportional hazards model suggested that age (all criteria), glaucoma type (criterion C), and preoperative IOP (criteria A and B) were associated with the surgical outcomes of BGI for OAG. The analysis for Kaplan–Meier survival curves indicated that the success probabilities for criteria B and C were higher in the older group than in the younger group. In addition, the IOP was lower in the older group 1 year after BGI, and the number of glaucoma medications tended to be consistently lower in the older group than that in the younger group. Furthermore, the multivariate Cox proportional hazards model revealed that older age is an independent factor associated with the surgical success of BGI for OAG in criteria B and C.

In this study, older age was a factor associated with the surgical success of BGI for OAG. Seven reports that have shown age as a factor associated with surgical outcomes of GDD were found^12–18^; three reports used BGI^12–14^, while the other four used AGV^15–18^. However, these reports included refractory glaucoma^12–18^. Generalizing the results from these reports to the surgical outcomes of BGI for OAG may be difficult because the surgical outcomes of GDD for refractory glaucoma are quite different from those of BGI for OAG. Among these seven reports, two studies reported opposite results to those in this study; Seah et al.^13^ and Tai et al.^17^ have reported that older age was associated with the surgical failure of GDD. In addition to the difference in glaucoma type (OAG and refractory glaucoma), the difference in the age range of patients between the two studies and this study may have caused the difference in the results. The age range was from 11 to 90 years in Seah’s study that included pediatric eyes and from 40 to 66 years in Tai’s study that did not include patients aged over 70 years. The age range in this study was from 43 to 91 years, reflecting the age distribution of patients with OAG. This study has the advantages of including only OAG eyes, using three criteria for surgical success, and using both univariate and multivariate analyses, which confirmed that age is an independent factor associated with the surgical outcomes of BGI for OAG. Interestingly, older age is associated with surgical success in this study which did not include childhood glaucoma, meaning that age ≥ 43 years still has the ability to predict the surgical outcomes of BGI for OAG eyes that did not have a developmental abnormality of aqueous humor outflow. Additional data also suggest the association between older age and surgical success of BGI for OAG eyes. The Tube Versus Trabeculectomy Study has shown that the cumulative probability of failure at 5 years after BGI was 42.9%, 44.6%, 38.3%, and 28.6% in patients aged < 60, 60–69, 70–79, and ≥ 80 years, respectively^9^. Although these data did not show statistically significant differences and included patients who underwent BGI or trabeculectomy, there was a tendency that older patients had a lower probability of failure. On the other hand, either glaucoma type in OAG (POAG and EXG) or preoperative IOP did not reach statistical significance in the multivariate analysis with the sample size of this study. Glaucoma type in OAG has not been independently associated with the surgical success of trabeculectomy in a previous report^20^. Previous studies including refractory glaucoma identified prior glaucoma surgery or prior incisional surgery^12,21–23^ and higher preoperative IOP^12,24^ as factors associated with the surgical outcomes of GDD surgery. The difference in glaucoma type (OAG and refractory glaucoma) could lead to a difference in the results because refractory glaucoma has stronger inflammation and conjunctival fibrosis than did OAG after surgery due to underlying diseases.

Less wound healing might be the mechanism of better surgical outcomes of BGI for OAG eyes in older patients because a clinicopathological study showed more extracellular matrix and activated fibroblasts in the non-functioning bleb than in the functioning bleb from GDD-inserted eyes of patients^25^. This suggests that active wound healing is associated with the surgical failure of GDD. In addition, fibroblasts play an essential role in wound healing, and their ability to regulate reactive oxygen species decreases with aging, resulting in less wound healing^26^. These results suggest that aging leads to less wound healing and contributes to the better surgical outcomes of BGI for OAG eyes.

Based on the results of this study, we should pay attention to the worse surgical outcomes of younger patients. To improve the surgical outcomes, attenuation of wound healing may be necessary. In the same concept as trabeculectomy, the intraoperative use and postoperative subconjunctival injection of antimetabolites such as MMC or 5-fluorouracil with GDD implantation have been studied to attenuate wound healing^27–29^. Two randomized clinical trials have shown that intraoperative MMC during AGV implantation did not improve the surgical outcomes^27,28^, suggesting that the nature of wound healing in GDD is different from that in trabeculectomy. On the other hand, a study that performed AGV implantation with both intraoperative injections of MMC and postoperative injections of 5-fluorouracil or MMC has shown a decreased hypertensive phase and improved surgical outcomes 1-year postoperation^29^. Thus, optimizing the intraoperative and postoperative use of antimetabolites might improve the surgical outcomes of BGI for younger patients with OAG.

There are limitations in the present study because of the retrospective nature. First, the method of the postoperative follow-ups was not unified. Second, the retrospective assessment of the medical records might be subject to selection bias. However, this was minimized in the present study by reviewing consecutive OAG patients who underwent BGI.

In conclusion, using the multivariate Cox proportional hazards model, this study demonstrated that older age is independently associated with a higher probability of success of BGI for OAG to achieve criteria B and C. Consistent with this, the probability of success is higher in the older group than in the younger group, and the postoperative IOP is lower in the older group 1 year after BGI. To advance the surgical strategies for OAG in an aging society, further research is needed on the mechanism of better surgical outcomes of BGI in older patients and the improvement of the surgical methods of BGI for younger patients with OAG.

## Methods

### Patients

In this study, the medical records were retrospectively reviewed for OAG eyes that underwent BGI surgery from November 2012 to April 2019 in Kumamoto University Hospital. The present study was approved by the Institutional Review Board of Kumamoto University. The Institutional Review Board waived the need for written informed consent because of the retrospective design of this study. Written informed consent was substituted by the opt-out method on our department website in which the study information was disclosed to the patients and the right for the patients to deny inclusion into this study was guaranteed. The protocol adhered to the tenets of the Declaration of Helsinki. The inclusion criteria were: patients aged ≥ 20 years, those with POAG or EXG, and eyes in which an IOP ≥ 20 mmHg was observed despite the use of glaucoma medication. The exclusion criteria were as follows: eyes that had no light perception, eyes that had a simultaneous cataract surgery at the time of BGI, and those who underwent a previous BGI or AGV surgery. Eyes having a history of ocular surgeries other than BGI or AGV surgery were not excluded. If both eyes from the same patient satisfied the inclusion criteria above, the first treated eye was included in this study.

### Surgical procedures

All BGI surgeries were conducted by one of three experienced surgeons (K.N., H.T., and T.I.). After sub-Tenon anesthesia, a conjunctival flap was created in the superotemporal or inferotemporal quadrant, depending on the previous surgical scar. Implant model BG101-350 (Abbott Medical Optics) was chosen in the eyes without a history of vitrectomy, while BG102-350 (Abbott Medical Optics) was selected in the eyes with a history of vitrectomy. To minimize the frequency of hypotony after BGI, the tube was completely occluded with 8-0 vicryl. The plate of the implant was placed under the rectus muscles and fixed to the sclera 8–10 mm behind the corneal limbus using 8-0 nylon. For the insertion of the tube into the anterior chamber, a tunnel of the sclera was made using a 23-gauge needle. If the eyes had a history of vitrectomy, the tube was inserted into the vitreous cavity via a tunnel made by a 20-gauge needle. The tube was sutured using 8-0 nylon to the sclera. Sherwood slits were made by the 8-0 nylon needle to minimize the postoperative IOP elevation. The tube was covered by a scleral patch graft or a half-thickness scleral flap. The conjunctiva was sutured by 8-0 vicryl. The patients received 1.5% levofloxacin for 1 month and 0.1% betamethasone for 3 months after BGI.

### Collected data

The collected preoperative information was as follows: the age at the time of surgery, gender, eye laterality, glaucoma type, best-corrected visual acuity (BCVA), IOP, the number of glaucoma medications, history of hypertension and diabetes mellitus, history of previous cataract surgery, the number of previous glaucoma filtration surgeries, and results of visual field test. The postoperative visits were usually scheduled 1 week, 1 month, 3 months, 6 months, 1 year, 2 years, 3 years, 4 years, and 5 years after BGI. The BCVA, IOP, complications, and number of glaucoma medications were evaluated at all postoperative visits. The IOP values were measured by a Goldmann applanation tonometer, and the mean of at least two consecutive measurements just before BGI was calculated for the preoperative IOP. Visual field was tested by a SITA-standard 24-2 program (Zeiss). When the eye had an advanced visual field defect, a SITA-standard 10-2 program was used. For statistical analysis, counting fingers were regarded as 1/100, hand motions were regarded as 1/800, light perception was regarded as 1/1600, and no light perception was regarded as 1/3200 based on the guideline^30^. The logarithm of the minimal angle of resolution was approximated by a logarithm of the reciprocal of the decimal BCVA. When the BGI needed to be removed, the subsequent IOP and glaucoma medication data were censored.

### Evaluation of surgical success and failure

Surgical success was defined as the following IOPs at two consecutive visits 3 months or later after surgery: 6–21 mmHg (criterion A), 6–18 mmHg (criterion B), and 6–15 mmHg (criterion C) without loss of light perception or additional glaucoma surgery. Hypotony (defined as IOP < 6 mmHg) was considered a surgical failure based on the guideline^30^. We defined cases of eyes needing surgery to control IOP (such as additional GDD surgery or cyclophotocoagulation) or BGI removal as a surgical failure. A surgical intervention due to complications was not considered a surgical failure if the IOP of the eyes was well-controlled after the intervention.

### Statistical analysis

The Mann–Whitney U test for continuous variables and Chi-square test or Fisher’s exact test for categorical variables were used for the comparison between two groups. For multiple comparisons within a group in Tables 4 and 5, the Wilcoxon signed-rank test with Bonferroni correction was chosen. The log-rank test was used to analyze the probability of success. The Cox proportional hazards model was employed for the univariate and multivariate analysis to determine the factors associated with the surgical outcomes. Factors with a *P* value < 0.20 by the univariate analysis were regarded as the candidate factors for the multivariate analysis. *P* values < 0.05 were considered statistically significant using the JMP (version 10.0.2, SAS Institute).

## Supplementary Information

Supplementary Information.

## Data Availability

The datasets generated during and/or analysed during the current study are available from the corresponding author on reasonable request.
