# Supplementary material for: Factors associated with the surgical outcomes of Baerveldt glaucoma implant for open-angle glaucoma, an age-related eye disease
Source: Sci Rep. 2022 Jan 25;12:1359. doi: 10.1038/s41598-021-04570-4 (PMC8789801; doi:10.1038/s41598-021-04570-4)
Supplement: Supplementary file 1 — Supplementary Information. [file 41598_2021_4570_MOESM1_ESM.pdf]

Supplementary Information

Factors associated with the surgical outcomes of Baerveldt glaucoma implant for open-angle glaucoma, an age-related eye disease

Satoshi Iraha, Yuji Takihara, Yui Urahashi, Takahiro Watanabe, Kenichi Nakamura, Mai Urahashi, Fumika Watanabe-Kitamura, Kei-Ichi Nakashima, Eri Takahashi, Sachi Kojima, Hidenobu Tanihara & Toshihiro Inoue

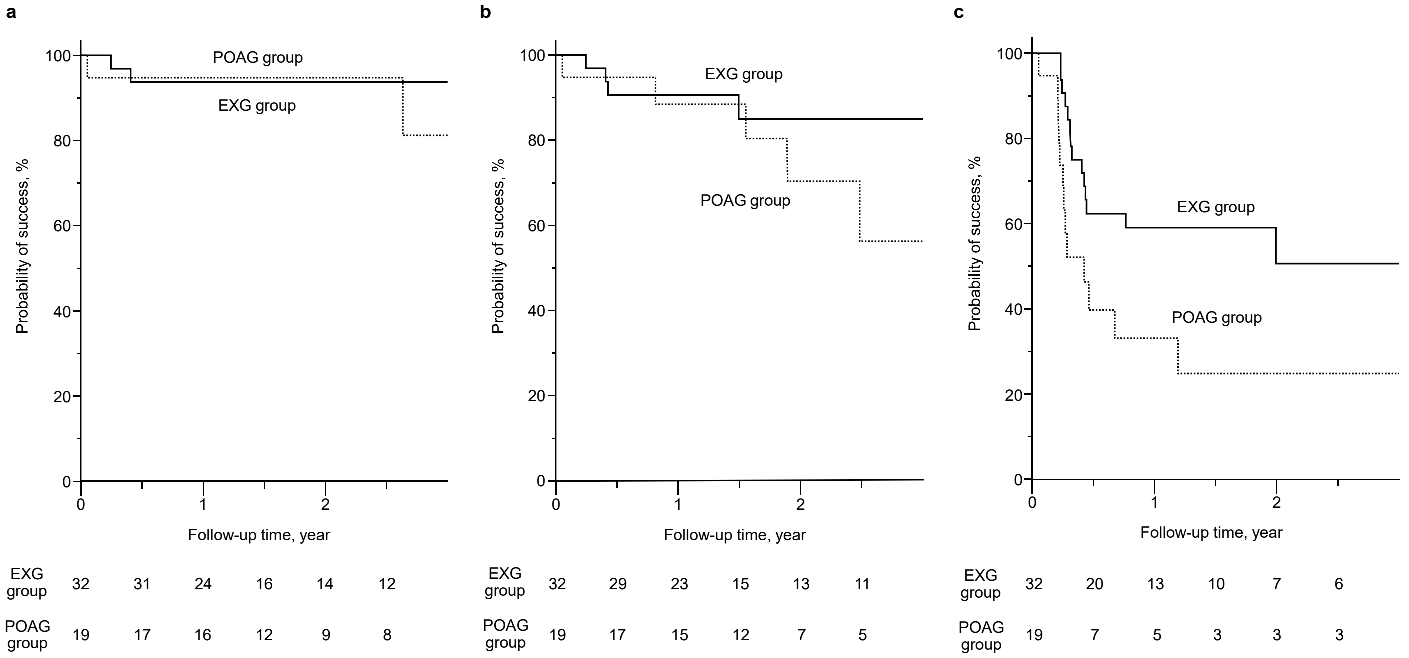

Supplementary Fig. S1. Comparison of the Kaplan–Meier survival curves for the success probability of BGI between POAG (dotted line) and EXG (solid line) groups. (a) Criterion A (6 ≤ IOP ≤ 21 mmHg), P = 0.59 by the log-rank test. (b) Criterion B (6 ≤ IOP ≤ 18 mmHg), P = 0.27 by the log-rank test. (c) Criterion C (6 ≤ IOP ≤ 15 mmHg), P = 0.03 by the log-rank test. The number of eyes at risk in each group is indicated at the bottom. BGI, Baerveldt glaucoma implant; POAG, primary open-angle glaucoma; EXG, exfoliation glaucoma; IOP, intraocular pressure.

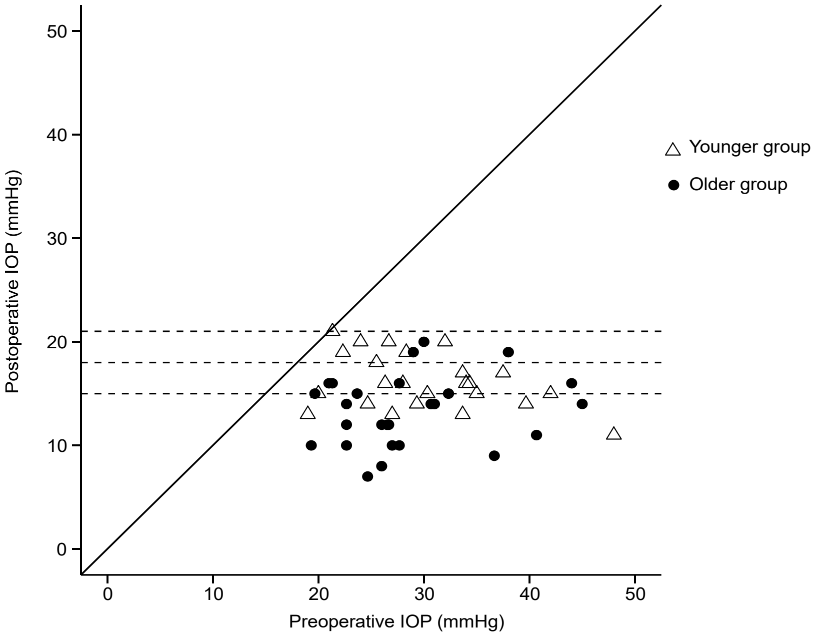

Supplementary Fig. S2. Scattergram comparing the preoperative IOP with the final postoperative IOP after BGI. Each eye is plotted as a single circle (older group) or open triangle (younger group). The dotted horizontal lines represent postoperative IOP levels of 21, 18 and 15 mmHg. IOP, intraocular pressure; BGI, Baerveldt glaucoma implant.

Complication	Early (onset ≤ 1 month)			Late (onset > 1 month)		
	Younger group (n = 25)	Older group (n = 26)		Younger group (n = 25)	Older group (n = 26)	
	No. (%)	No. (%)	P value	No. (%)	No. (%)	P value
Shallow or flat anterior chamber	6 (24%)	2 (8%)	0.25[a]	1 (4%)	1 (4%)	1.00[a]
Choroidal detachment	7 (28%)	8 (31%)	0.69[b]	1 (4%)	2 (8%)	1.00[a]
Hypotony maculopathy	0	1 (4%)	1.00[a]	1 (4%)	0	0.49[a]
Cystoid macular edema	0	0	NA	1 (4%)	0	0.49[a]
Hyphema	9 (36%)	7 (27%)	0.48[b]	0	0	NA
Tube corneal touch	1 (4%)	3 (12%)	0.61[a]	0	1 (4%)	1.00[a]
Tube iris touch	4 (16%)	2 (8%)	0.42[a]	0	0	NA
Tube occlusion	1 (4%)	0	0.49[a]	0	1 (4%)	1.00[a]
Sclera patch exposure	1 (4%)	0	0.49[a]	0	0	NA
Blebitis suspected	1 (4%)	0	0.49[a]	0	0	NA
Wound leak	2 (8%)	1 (4%)	1.00[a]	0	0	NA

Supplementary Table S1. Postoperative complications. [a]Fisher's exact test. [b]Chi-square test.
